# Aging and Herbal Interventions: Mechanistic Insights and Therapeutic Potential

**DOI:** 10.1111/jocd.70335

**Published:** 2025-07-24

**Authors:** Divyesh Suvedi, Anand Kumar, Sonika Kalia, Arun Kumar, Sapna Koul, Abija James, Deepak Kumar, Rupak Nagraik, Henok Gulilat

**Affiliations:** ^1^ Faculty of Applied Sciences and Biotechnology Shoolini University Solan Himachal Pradesh India; ^2^ Department of Pharmaceutical Chemistry, School of Pharmaceutical Sciences Shoolini University Solan Himachal Pradesh India; ^3^ Department of Biotechnology, School of Applied and Life Sciences Uttaranchal University Dehradun India; ^4^ Polish Academy of Sciences Institute of Biochemistry and Biophysics Warsaw Poland; ^5^ Department of Biotechnology Graphic Era (Deemed to Be University) Dehradun India; ^6^ Department of Biomedical Sciences, Institute of Health Jimma University Jimma Oromia Ethiopia

**Keywords:** clinical trials, herbal extracts, nano‐formulations, natural cosmetics, skin aging

## Abstract

**Background:**

Skin aging is a multifactorial process influenced by an individual's genetic predisposition, lifestyle choices, and environmental factors. This process leads to epidermal thinning, a reduction in collagen and elastin levels, and a decrease in skin suppleness. While aging cannot be entirely prevented, its effects can be mitigated through the application of appropriate skincare products.

**Aim:**

This review examines the potential of natural herbal extracts in anti‐aging skincare formulations, focusing on their bioactivity and safety, which aligns with current consumer trends favoring natural, organic, and eco‐friendly products.

**Methods:**

A systematic literature review was performed with the help of PubMed and ScienceDirect to thoroughly examine articles between 2000 and 2025. The review made use of key phrases including “skin aging,” “herbal extracts,” and “anti‐aging cosmetics.”

**Results:**

Bioactive compounds such as polyphenols, flavonoids, and essential oils, present in plants, confer multiple benefits for the skin, including moisturizing properties, barrier repair agents, antioxidants, sunscreens, and anti‐inflammatory effects. These compounds also protect and rejuvenate the skin through distinct anti‐aging mechanisms.

**Conclusion:**

The use of herbal extracts in combination with nano‐formulations enhances the efficacy of skincare regimens while improving stability. The integration of plant‐based resources, when combined with a balanced diet and a healthy lifestyle, can significantly enhance holistic and effective anti‐aging skincare.

## 1 Introduction

Aging is an unavoidable phase of life, which is a multifactorial transition process, like biological changes, that is characterized by the progressive decline of physiological activity and the increased vulnerability to age‐related disorders like osteoporosis and Alzheimer's. It first begins at the cellular level and continues throughout the lifespan, ultimately turning into death [1]. Aging shows across biological factors, which are the most visible factors during aging, followed by psychological and social dimensions. This includes a marked reduction in immune competence, deterioration of musculoskeletal integrity, and sensory impairments [2, 3, 4]. The process is based on intrinsic (genetic and metabolic) and extrinsic (lifestyle‐ and environmental‐related) factors. Intrinsic aging comprises mechanisms like impaired collagen synthesis, mitochondrial decay, hormonal changes, and cell senescence. On the other hand, extrinsic aging, more simply known as photoaging, is significantly determined by ultraviolet (UV) radiation, pollution in the environment, unhealthy dietary practices, smoking, and psychological tension. These accelerate the production of reactive oxygen species (ROS) and thus oxidative stress, lipid peroxidation, protein crosslinking, and the production of advanced glycation end products (AGEs), which all lead to evident signs of aging such as wrinkles, pigmentation, dryness, and loss of elasticity of the skin [3, 5].

In ancient times, anti‐aging treatments generally focused on the skin, which is the most outward organ of the body and showed the most affected result of aging. Nowadays, science has evolved, and we are using topical chemical agents, cosmetic treatments like chemical peeling, laser resurfacing, microdermabrasion, and invasive treatments such as botulinum toxin (Botox) injections and dermal fillers [6]. While effective in providing immediate cosmetic results, they also have very severe limitations, such as high price, potential side effects (e.g., skin redness, allergic contact dermatitis, post‐inflammatory hyperpigmentation), and scarce long‐term safety evidence. Additionally, many of these chemical‐based skincare products contain synthetic additives and preservatives, raising public health and environmental concerns. The awareness has shifted consumer interest toward safer, more sustainable solutions that provide long‐term skin health benefits without compromising [7].

In response to these concerns, the global anti‐aging market has experienced rapid growth, reaching an estimated value of $216 billion in 2021 [7]. This growth is fueled by awareness and a growing global aging population. Although the traditional primary market has been middle‐aged and older consumers, younger age groups—namely Millennials (25–40 years) and Generation Z (under 25 years)—are increasingly embracing pre‐aging. These consist of early and regular application of sunscreen, antioxidants, and lifestyle changes to retard the onset of visible signs of aging. Men are also increasingly taking part in skincare, a sign of changing gender norms and wider acceptance of male grooming routines. In addition, the need for natural, plant‐derived, and “clean” skincare products is increasing as consumers are looking for products that meet safety, sustainability, and ethical sourcing standards [8, 9].

Natural products, particularly those from medicinal plants, have been recognized as viable alternatives to artificial cosmetics in anti‐aging treatments. These ingredients usually contain plant extracts, essential oils, vitamins (e.g., A, C, and E), and minerals with antioxidant, anti‐inflammatory, and photoprotective activities [10]. The drug efficacy of plant ingredients stems from their richness in bioactive compounds, including flavonoids, phenolics, and terpenoids, which can neutralize free radicals, induce collagen synthesis, and aid in epidermal barrier function [11]. These compounds not only act against environmental insults but also regulate intracellular signaling pathways that are implicated in skin repair and regeneration. For example, green tea polyphenols, grape resveratrol, and turmeric curcumin are extensively documented for their reactive oxygen species scavenging activity as well as matrix metalloproteinases inhibition, enzymes responsible for the degradation of collagen and elastin in the dermis [12].

By the World Health Organization's account, more than 20 000 medicinal plant species are used in 91 nations and represent an immense pool of botanical ingredients available for cosmetic use. Preparations based on herbal extracts are viewed as milder, more compatible with skin physiology, and generally less likely to cause adverse reactions than synthetic alternatives. Furthermore, blending herbal and chemical ingredients is being researched more and more to synergize the strengths of ancient and conventional systems. These hybrid products may improve product performance while satisfying consumer requirements for transparency and natural character [12]. Advances in formulation science, such as nanoencapsulation, emulsification, and transdermal delivery systems, have further enhanced the stability and bioavailability of plant‐derived materials in cosmetic products.

This review gives a comprehensive overview of plant‐derived active ingredients used in anti‐aging cosmetics. It discusses their functions in water retention, brightening of the skin, photoprotection, regeneration of the epidermis, and regulation of inflammation. The review also critically evaluates the strengths and shortcomings of natural cosmetics in relation to standard chemical products. It highlights the growing trend of incorporating herbal extracts into current chemical‐based cosmetics in order to obtain a higher level of therapeutic effects. Finally, the review emphasizes the need for ongoing research into herbal extract standardization, safety, and clinical efficacy to assist in developing useful, science‐based natural anti‐aging remedies.

## 2 Properties of Herbal Extracts for Anti‐Aging

### 2.1 Moisturizing

Moisturization serves as a first line of defense against skin aging, helping to maintain the skin's elasticity and appearance while strengthening the barrier against environmental stressors [13]. The Stratum Corneum (SC) is a water‐lipid membrane that protects the body from external factors. For the SC to function optimally, it must contain around 30% water, which supports desquamation and serves as a critical barrier against infection [14]. Natural moisturizing factors within the SC contribute to hydration and water retention. When the moisture levels in the skin go below 10%, it typically becomes rough and dry and can even scale finely [15]. Moisturizers can help repair damaged SC. Water‐soluble ingredients provide temporary hydration by diffusing into the epidermis, while fat‐soluble ingredients form a barrier film over the skin's surface, reducing transepidermal water loss (TEWL) [16]. Additionally, lipids and other ingredients can interact with the epidermal lipids, providing relief from the dryness of the skin. Plant extracts are rich in two types of moisturizing agents. Polysaccharides and glycosides contain hydroxyl groups that retain water through hydrogen bonding, while vegetable oils improve the barrier functions of the skin, stimulate SC regeneration, minimize water loss, and influence sebaceous gland activity [17].

**FIGURE 1:**
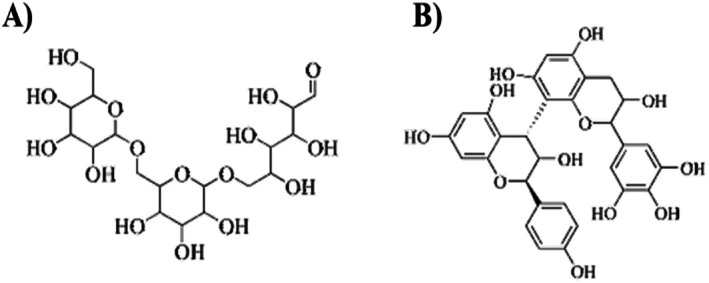
(A) Dextran plant polysaccharide. (B) Proanthocyanidins.

Most plant polysaccharides, like dextran, exhibit humectant properties, as shown in Table 1. This is largely dependent on the content, glycosyl structure, and molecular weight of the polysaccharides. Polar carboxyl groups found in the structures can form hydrogen bonds with the water molecules to retain moisture in the stratum corneum even at low concentration levels (Figure 1A). Acetylated mannan, the primary active component in *Aloe barbadensis miller* extract, is commonly used in cosmetics for its wound‐healing properties and ability to retain moisture. Alginate, obtained from brown algae, is used as an emulsifier as well as a moisturizer. Alginate absorbs ambient moisture and develops a protective structure for decreasing the loss of water from the skin by a great softening action on the skin. These include *Opuntia dillenii, astragalus, Pholiota nameko*, and *Dendrobium officinale*, among other water‐soluble plant polysaccharides, which exhibit excellent properties of water absorption [7, 24].

**TABLE 1:** Polysaccharides with a moisturizing effect.

Polysaccharide	Primary active element	Function in cosmetics	References
*Aloe barbadensis miller* polysaccharide	Acetylated Mannan	Moisturizing, skin wound healing	[18]
Alginate	Brown algae polymer	Moisturizing, stabilizing	[19]
*Opuntia dillenii* polysaccharide	*Opuntia dillenii*	Water absorption	[20]
*Astragalus* polysaccharide	*Astragalus*	Water absorption	[21]
*Pholiota Nameko* polysaccharide	*Pholiota Nameko*	Water absorption, moisturizing	[22]
*Dendrobium Officinale* polysaccharide	*Dendrobium Officinale*	Water absorption	[23]

Among the two primary bioactive compounds within green tea, highly prized for their uses in skincare with benefits of notable health, include tea polyphenols (TPP) and tea polysaccharides (TPS). TPP reduces lipid peroxidation within cells by increasing glutathione peroxidase levels and neutralizing free radicals, thereby protecting cells from oxidative damage [25]. Additionally, TPP promotes cell proliferation, strengthens the skin barrier, accelerates wound healing, and improves fluid flow within skin tissue. By reducing intercellular viscosity, it enhances skin elasticity. All catechins are present in green tea, but epigallocatechin‐3‐gallate (EGCG) is one of the most active due to its potent free radical scavenging ability and its capacity to significantly inhibit the action of ROS by having high antioxidant potency. Another key green tea constituent, proanthocyanidins, is a hydrophilic polyphenolic polymer with phenolic hydroxyl groups. It enhances moisture retention in the stratum corneum, leading to improved hydration and skin elasticity, as shown in (Figure 1B). This resulted in a moisturizing effect meant to enhance the hydration and elasticity of the skin [26]. These compounds offer significant benefits in skincare formulations, particularly due to their antioxidant properties. As a result, green tea bioactives hold great promise for modern cosmetic formulations, offering enhanced skin health, improved barrier function, and increased resistance to environmental stressors.

This *chamomile* extract, rich in quercetin, displays impressive effects on the skin: it improves moisture retention, promotes repair in the skin, and helps control sebum excretion while lowering irritability. Quercetin is profoundly involved in this process by stimulating tight junction proteins for stronger cell‐to‐cell adhesion and strengthening the barrier function of the skin, which is useful in the regulation of water and minimizes dehydration as well as reactivity [27]. Similarly, there is a high number of flavonoids still displayed as some of the most effective moisturizers in cosmetic products, for example, chrysanthemum, pear cactus, and bamboo; the deep moisturizing of these plant extracts gives wonderful hydration to the skin [7].

*Centella asiatica* extract is another leading natural moisturizer boasting wound‐healing and moisturizing properties. The pentacyclic triterpenoid saponins, more importantly, asiaticoside, increase hydration of the skin and are thus commonly applied in products like wipes and skincare creams and ointments to heal wounds and scars. Hyaluronic acid is also a significant source of hydration for skin as it is of high molecular weight and viscous, which enables it to retain a large amount of water for enhanced moisturization. This attribute makes hyaluronic acid be at the peak that crowns it as the gold standard concerning the preservation of hydration and prevention of moisture loss in the cosmetic industry [28].

Vegetable oils are commonly applied in creams, lotions, and conditioners in cosmetics that utilize unsaturated fatty acids (as shown in Table 2). For example, wheat germ oil gives an armor layer on the skin, assists in protecting it from moisture loss, and promotes the regeneration of skin qualities, making it ideal for dry, rough‐surfaced skin [35]. Emollient softening *Cocos nucifera* oil increases aquaporin AQP3, filaggrin, and integrin expression to enhance keratinocyte differentiation [36]. More than that, seed extracts of *Hippophae rhamnoides L*. and *Rubus idaeus* are emollients that maintain hydration on the skin through stratum corneum strength and cell structure protection [37]. In addition to hydrating the skin, these plant‐based emollients play a fundamental role in strengthening and supporting the skin, hence acquiring a status that is considered vital in cosmetic formulations.

**TABLE 2:** The function of vegetable oils in cosmetics.

Vegetable oils	Source	Family	Function	References
(Wheat germ oil)	*Triticum vulgare*	Poaceae	Promotes skin regeneration and forms a protective layer	[29], [30]
	*Triticum aestivea*			
(Coconut oil)	*Cocos nucifera*	Arecaceae	Work as a moisturizer increases expression levels of filaggrin and integrin, aids in keratinocyte differentiation, and aquaporin AQP3	[31]
(Sea buckthorn oil)	*Hippophae rhamnoids L*.	Elaeagnaceae	Moisturizes, protects, regenerates, and softens the stratum corneum, ensuring structural stability between skin cells	[32], [33]
	*Hippophae salicifolia*			
(Raspberry oil)	*Rubus*	Rosaceae	Exhibits moisturizing effects, protects, and promotes regeneration	[34]

### 2.2 Barrier Repair

**FIGURE 2:**
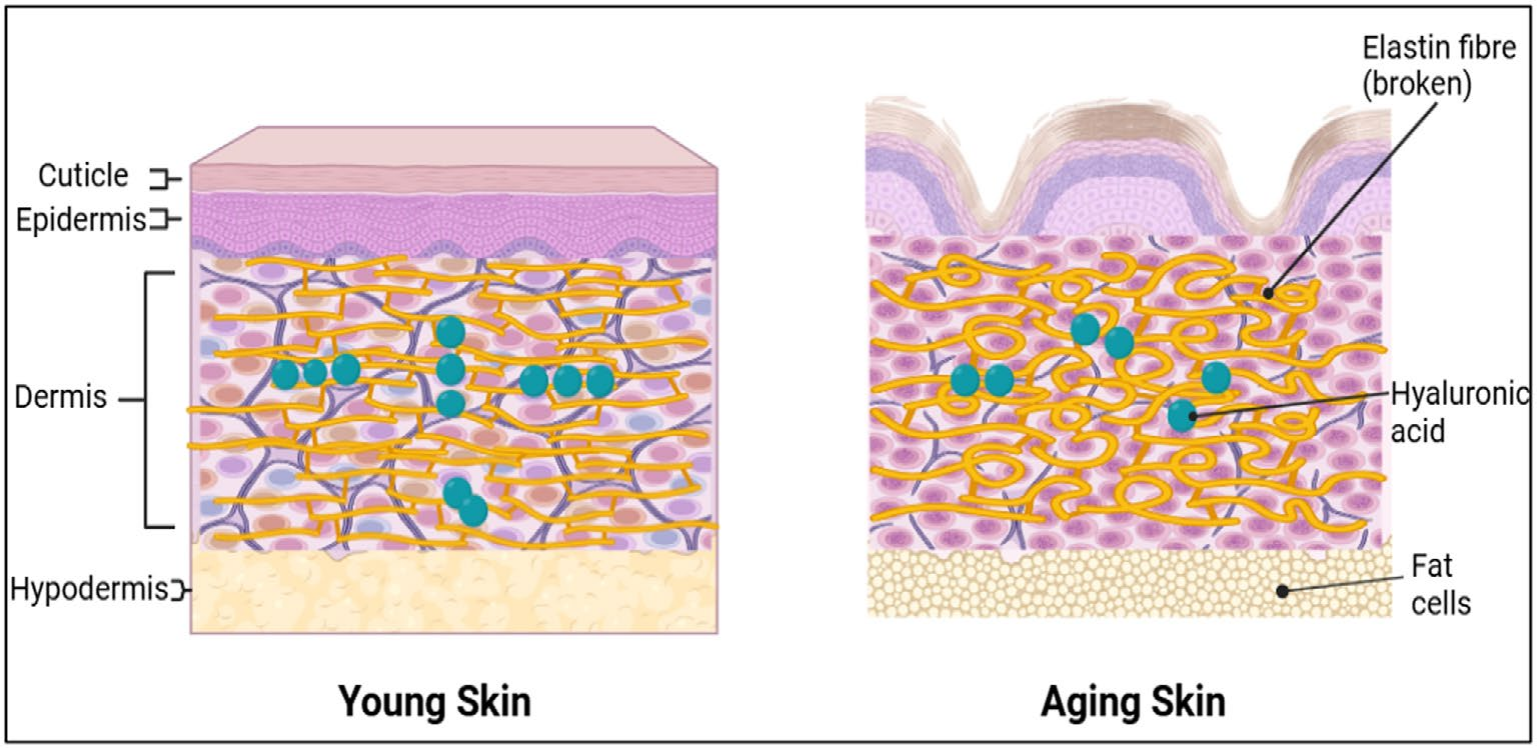
The difference between young and aged skin in terms of elasticity is due to shattered elastic fibers, less collagen, and hyaluronic acid.

Aging in humans and other organisms is marked by the gradual accumulation of advanced glycation end products (AGEs) in the body. Common AGEs found in the skin include pentosidine, carboxymethyl‐lysine, carboxyethyl‐lysine, methylglyoxal, glyoxal, fructose‐lysine, and glucosepane. AGE receptors are primarily located in the epidermis and dermis, with significantly higher expression levels observed in sun‐exposed skin compared to areas shielded from sunlight. This suggests that UV exposure intensifies AGE accumulation, accelerating skin aging through oxidative stress and glycation processes [38] (See Figure 2).

The maintenance of healthy, intact skin is crucially dependent on the presence of a functioning skin barrier. When the barrier function is disrupted, there can be excessive water loss through the transepidermal route, sensitivities of the skin, and susceptibility to environmental aggressors. Over the years, there has been considerable interest in plant extract cosmetics that can restore and strengthen the barrier function of the skin [39]. Plant extracts with high levels of bioactive compounds are potential candidates for replacing synthetic alternatives in natural skin care products for consumers who seek softer, gentler products. Such examples include *chamomile* extract, which improves the skin barrier and reduces irritation, along with rehydrating the oil balance [40]. Its constituents, in particular quercetin, aid in strengthening junction‐binding proteins to improve cellular connections and, consequently, improve barrier function [41].

Another ingredient extremely active in botanicals is *Centella asiatica* extract, which features excellent wound‐healing and regenerative properties [42]. The main active compound of the extract, madecassoside, is used in the majority of cosmetic formulas due to its ability to enhance skin regeneration and minimize scarring, so it should feature in any product focusing on wound and scar treatment [43]. Hyaluronic acid is naturally found in the skin and functions to retain moisture within it. Cosmetically, it retains water molecules in the matrix of the skin so that the integrity of the skin's barrier remains intact. Hydration keeps the skin moist and lifted, reduces dehydration, and improves the elasticity of the texture of the skin [44].

Several vegetable oils have been found to play significant roles in barrier repair formulations. The large category of major botanical oils with a selective capacity to create an occlusive skin barrier, restore the amount of moisture, and promote regeneration of skin includes wheat germ oil, coconut oil, sea buckthorn oil, and raspberry oil [45]. Together, these oils fortify the stratum corneum, the outermost skin layer, against environmental stress. Plant extract cosmetics vow to heal and rejuvenate the skin barrier in general since they reveal a holistic approach to skincare based on taking advantage of the multiple benefits that natural botanicals are blessed with through both functional and cosmetic improvements in skin health.

### 2.3 Antioxidant Activity

**FIGURE 3:**
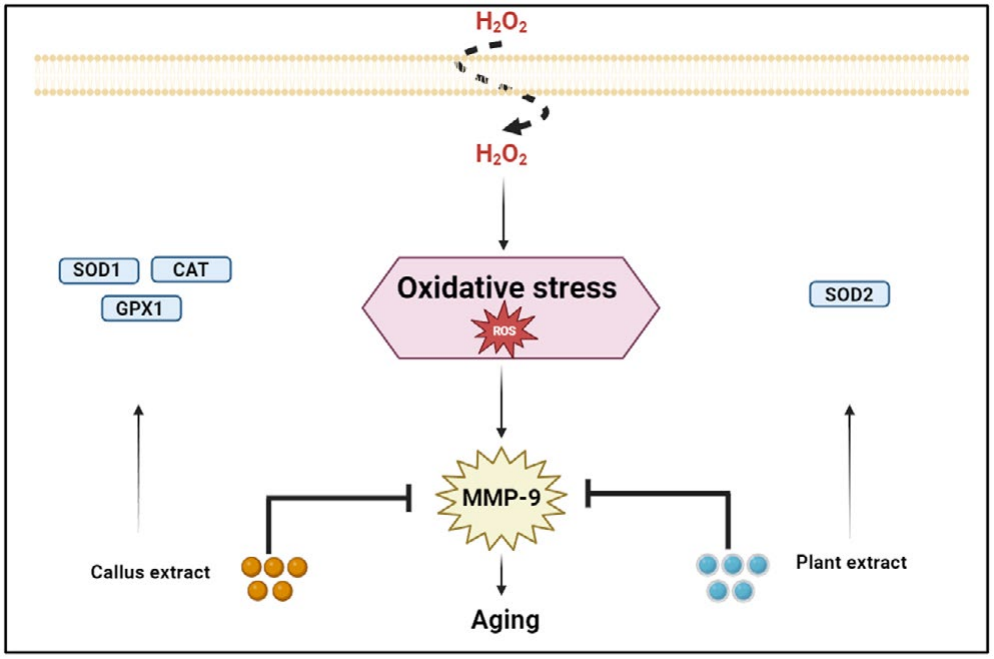
The mechanism shows the antioxidant and anti‐skin aging activities of *Centella asiatica* extract and callus extract.

Oxidation is one of the most natural metabolic processes that occur in the human body, and it results in the creation of a massive amount of free radicals and reactive oxygen species. When the antioxidant defense system fails to scavenge such excess free radicals, oxidative stress emerges [46]. Such an imbalance can further lead to several skin aging problems caused by ROS, which are superoxide radicals, hydrogen peroxide, hydroxyl radicals, singlet oxygen, and nitric oxide. These biologically active species can cause DNA mutations, modify gene expressions, and degrade such integral cellular constituents as lipids and proteins [47]. Furthermore, ROS activates such degradative enzymes as collagenase, elastase, superoxide dismutase (SOD1), and hyaluronidase for facilitating the breakdown of extracellular matrix components, hence promoting premature aging skin. In vitro, the phytochemical extract of *Centella asiatica* has exhibited potential antioxidant and inhibitory activities against some destructive enzymes, as shown in Figure 3.

Among the naturally occurring antioxidants, polyphenols have been recognized as the most abundant and powerful free radical scavengers because of their hydroxyl groups attached to their aromatic rings. They are mainly categorized into flavonoids, phenolic acids, stilbenes, tannins, and coumarins. Notably, proanthocyanidins are the antioxidant agents of highest activity throughout the plant kingdom [48]. Hydroxytyrosol, resveratrol, curcumin, and rosemary extract possess significant antioxidant properties. These can shield skin cells from the oxidation of DNA, further preventing UV‐induced mutagenesis [49]. Of the compounds mentioned above, the resveratrol present in red wine is essentially valued for its anti‐aging attribute, thereby fitting into most antioxidant‐enriched cosmetic formulations [50].

### 2.4 Antioxidant Flavonoid Pathways in Aging

Flavonoids are naturally occurring phenolic substances found in fruits, vegetables, tea, and other plant foods and are most popularly known as potent antioxidants with broad protective powers for cells and skin [48]. The antioxidant action of flavonoids is primarily attributed to their ability to scavenge reactive oxygen species (ROS) through the donation of hydrogen atoms or electrons from hydroxyl groups that are on the B‐ring of their molecular structure [51]. This stabilizes free radicals, such as hydroxyl and peroxyl radicals, blocking oxidative damage to cellular components. Besides directly scavenging ROS, flavonoids suppress the generation of these toxic species by inhibiting the activity of crucial enzymes responsible for ROS production, including xanthine oxidase, NADPH oxidase, and mitochondrial succinoxidase. Flavonoids like quercetin and luteolin are highly effective in inhibiting these enzymes, thus preventing oxidative stress in various cellular locations [52].

Another important mechanism is metal ion chelation. Catechol‐containing flavonoids can chelate redox‐active metals such as iron and copper, which act as catalysts in Fenton reactions that result in the formation of highly reactive hydroxyl radicals. Chelating these metals with flavonoids inhibits further oxidative damage. In addition to their direct antioxidant actions, flavonoids regulate cellular defense systems through the activation of signaling pathways, including the Nrf2‐ARE pathway. Upon activation, Nrf2 moves to the nucleus and binds to the antioxidant response element (ARE), leading to the induction of endogenous antioxidant enzymes like superoxide dismutase (SOD), catalase (CAT), and glutathione peroxidase (GPx) [53].

Hydroxy acids consist of alpha‐hydroxy acids such as citric, glycolic, lactic, malic, tartaric, and amygdalic acids. They are applied as topical exfoliants and skin rejuvenators [54]. The high penetration capability of these acids makes them easily absorbed in the stratum corneum by providing stimulation to higher turnover of cells, breaking down the intercellular bonds and being useful in the removal of photoaging, fine wrinkles, acne, scars, and pigmented conditions of the skin [55]. Other cosmetic formulations include several vitamins such as ascorbic acid, vitamin C; tocopherol, vitamin E; retinol, vitamin A; nicotinamide, vitamin B3; α‐lipoic acid; and coenzyme Q10, all of which are antioxidants. These vitamins have been major contributors to the reduction of oxidation, stimulation of synthesis of collagen, maintenance of the vitality of the skin, and upon addition to creams, these result in the neutralization of oxidative stress, increase the resilience of the skin, and result in healthier‐looking, younger skin (Table 3) [56].

**TABLE 3:** Antioxidant properties of vitamins.

Vitamin	Properties and benefits
Vitamin C (VC)	Enhances antioxidant effects and collagen stability, promotes skin firmness, and reduces fine lines and scars
Vitamin E (VE)	Effective antioxidant that counters free radicals, softens skin, prevents oxidative damage, and maintains skin barrier function
Vitamin A (VA)	Stimulates new skin cell generation, promotes collagen production, and improves wrinkles, scars, burns, and stretch marks
Vitamin B3	Preserves skin moisture, balances oil secretion, treats solar keratosis, and minimizes fine lines
α‐Lipoic Acid	Potent natural antioxidant, combats various free radicals, known as a universal antioxidant, found abundantly in plants
Coenzyme Q10	Reduces free radical production and diminishes DNA damage in keratinocytes

These vitamins provide multiple benefits for the skin, including enhancing collagen synthesis, providing antioxidant protection, and maintaining hydration. Certain vitamins can be combined to enhance their effectiveness in skincare formulations; for example, vitamins C and E are often paired for greater impact. Additionally, α‐lipoic acid and coenzyme Q10 contribute to antioxidative defense by minimizing environmental stressors, such as UV light, that can damage the skin [57, 58]. Their importance in preserving the health and radiance of the skin is underscored by their widespread use in cosmetic products.

### 2.5 Sunscreen and Pigmentation

Photoaging is caused by increased exposure to UV radiation. Today, it is common for skincare companies to include UV‐scattering and absorbing agents in their anti‐aging cosmetic products as a defense mechanism against damage from UV light. Conversely, UV light induces matrix metalloproteinases, which contain elastases that deconstruct collagen, fibronectin, and elastin out of the extracellular matrix, thus accelerating the aging of the skin [59]. Long‐term exposure to the sun has also been proven to be associated with melanoma and nonmelanoma skin cancers. In preventing photoaging, one of the main preventions is the avoidance of direct exposure to the sun and the use of protective clothing, hats, sunglasses, and sunscreen.

Photoaging accounts for about 80% of the aging evident on the faces of people and is primarily caused by the effect of cumulative exposure to ultraviolet radiation and the latter's effect on the pigmentation in the skin. There are three kinds of UV radiation: UVA, UVB, and UVC, with a wavelength range between 320 and 340 nm, 290 and 320 nm, and 200 and 280 nm, respectively. All types of UVA and UVB cause DNA damage and have the potential to result in mutations, cell death, or malignant transformation [60]. The amount of the product, ROS, released increases with photoaging and natural aging. Oxidative stress within a cell is initiated by elevated levels of ROS, destroying nucleic acids, proteins, and lipids and thus accelerating both aging of the skin and pigmentation.

Most whitening products target a decrease in the amount of melanin, a pigment that may cause color in the skin. These decreases result from the inhibition of an enzyme called tyrosinase, which plays a role in the process of production and, more importantly, in differentiating melanocytes in the development and maturation process [61]. Active agents in cosmetic sunscreens are mainly active through UV light absorption. Skin‐whitening treatment, however, is meant to suppress the synthesis of melanin; this is achieved by reducing the activity of tyrosinase. These whitening agents have been reported to act as a competitive inhibitor of tyrosinase and could prevent it from either maturing or becoming mobile within cells [62].

However, the long‐term application of traditional whitening agents, among which are steroids, hydroquinone derivatives, arsenic (As) and mercury (Hg) compounds, can lead to hazardous, severe health issues. Hydroquinone is a skin‐lightening cream that, with long‐term use, can cause Ochronosis, a skin disorder characterized by blue‐black pigmentation. Additionally, prolonged and excessive exposure to toxicants such as mercury (Hg) and arsenic (As) in cosmetics can lead to skin damage, including rashes, irritation, and discoloration. These substances have also disrupted DNA and RNA functions and caused renal and neurological dysfunction, cataracts, glaucoma, impairment of fetal development in pregnant women, and inhibition of tyrosinase activity, resulting in decreased pigmentation. Other potential health effects include gastrointestinal problems, cancer, hepatotoxicity, nephrotoxicity, and damage to the central nervous system [63, 64]. In contrast, organic sunscreens contain P‐aminobenzoic acid and dibenzoyl methane, two compounds approved by the US FDA to protect against UV radiation without causing undesirable effects, making them reliable sunscreen agents for skin (Ren et al. 2021). Other plant chemicals with a benzene ring or conjugated structure can also have an organic sunscreen‐like action to protect against UV radiation naturally [65].

Arbutin, derived from wheat and azalea leaves, is an excellent UV absorber and tyrosinase inhibitor that reduces melanin buildup. Undecylenic acid ester of arbutin is a prodrug, as it increases arbutin's stability along with enhancing its skin penetration and inhibiting tyrosinase and phenoloxidase enzymes [66]. Licorice, due to its flavonoid content, is a whitening agent and sunscreen due to its UV‐absorbing characteristics [67]. Some of the active ingredients used in natural sunscreens also contain anthocyanins that inhibit UV damage to plants through DNA repair and scavenging oxidation. Lignin is a form of natural sunscreen because it neutralizes the hydroxyl radicals in combination with other active ingredients. Salidroside is an active ingredient extracted from *Rhodiola rosea* that has antioxidant properties while having tyrosinase inhibitory activity and thus prevents damage through UV rays [68]. Green tea polyphenol extracts that contain epicatechin 3‐O‐gallate and catechin 3‐O‐gallate can diminish the threats that UV‐induced immunosuppression and damage impose on the skin. Vitamin C, or ascorbic acid, has a crucial role in the prevention of melanin formation and the whitening stabilization of skin [69].

### 2.6 Anti‐Inflammatory Activity

Skin irritation and aging are among the related factors. The development of aging can even be sped up by prolonged irritative inflammation. Inflammatory diseases provoke the development of cytokines, free radicals, and other destructive substances that can damage cellular structures and disrupt the normal synthesis and function of collagen and elastin. Moreover, inflammation diminishes the natural barrier function of the skin, causing loss of moisture and structural changes in the cells of the skin, which makes the pace of aging of the skin speed up even more. Hence, controlling inflammation of the skin is very important to avoid and delay the process of aging. Related studies have shown that para‐inflammation in the retinas of elderly subjects is caused by free radicals and oxidized lipoproteins. Such para‐inflammation appears in several different forms; by using microarray analysis combined with complementary system components, researchers found the molecular overexpression of various genes involved in immune response and inflammation. These include pro‐inflammatory enzymes, cytokines, chemokines, as well as components of the complement system [70]. “Inflammatory aging” can be defined as a slow, progressive, low‐grade, sterile inflammation, which usually accompanies aging and is closely related to immunosenescence—the aging of the immune system [71]. Interestingly, two earlier studies have reported that inflammation, mainly by TNF, plays a more significant role in the alterations of gut microbiota with age, suggesting that the modifications in gut microbiota are more related to the inflammatory process rather than to age‐related phenomena [72].

**FIGURE 4:**
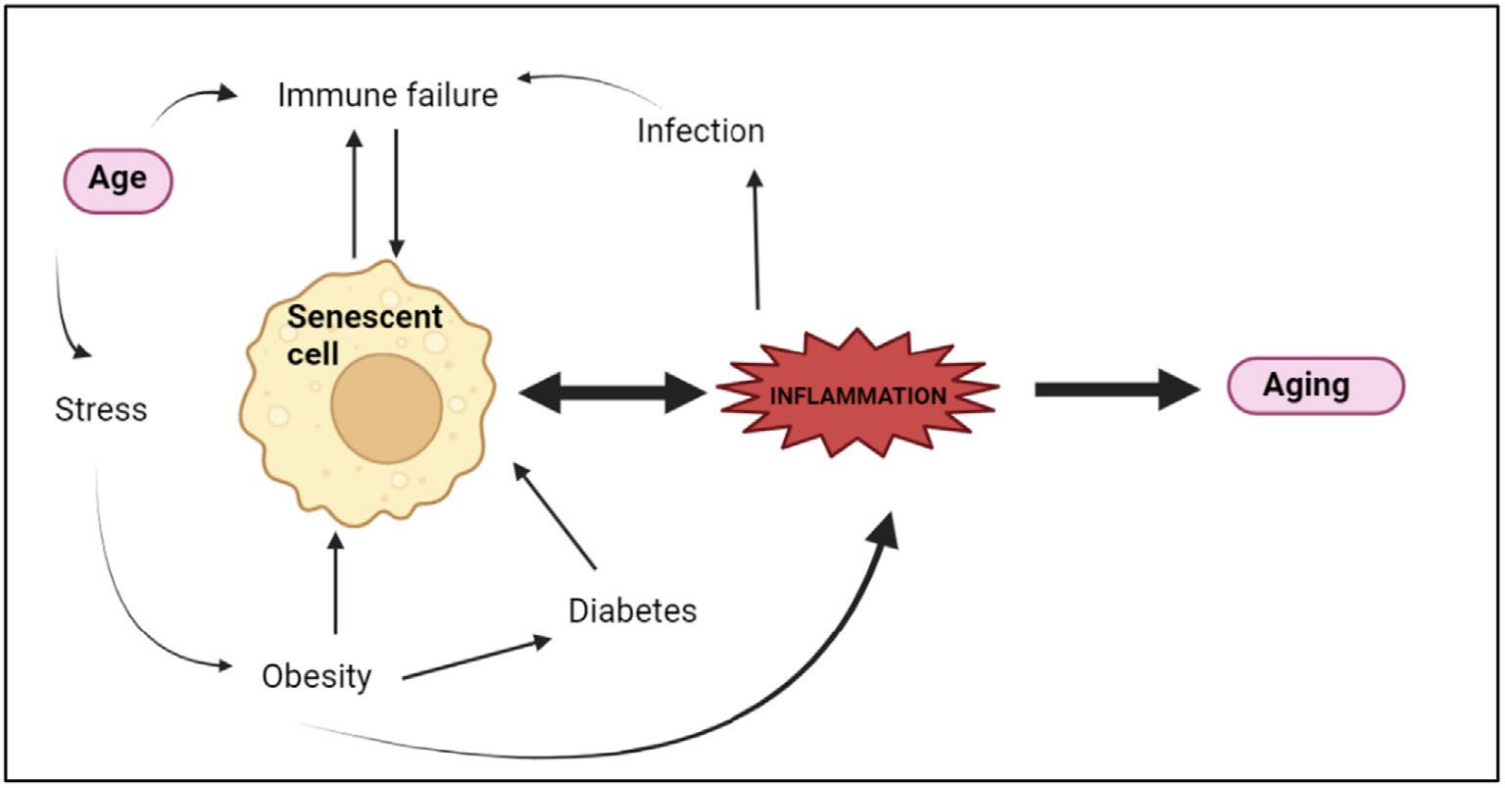
A key feature of aging and inflammation in senescent cells.

In other words, the contribution of senescent cells in the aging of the skin is very important. These cells create pro‐inflammatory factors and growth factors that cause inflammation, cell proliferation, and modification in pathways for cell death. This occurs when these cells accumulate in the skin, causing problems during old age, such as wrinkling, stress, obesity, infection, dysfunction of the immune system, and loss of firmness and elasticity. These results are due to the failure of cells in the aging process through telomere shortening, DNA damage, and oxidative stress. The cells in immune senescence pose a significant problem in the overall removal from the body, as they compromise immunity at large. Several factors, such as obesity, diabetes, hyperglycemia, and metabolic stress, lead to cellular senescence due to increased inflammation. Viral and bacterial infections also accelerate the aging process to a great extent (as shown in Figure 4). Older cells could send signals to the surrounding cells through pro‐inflammatory signals that are associated with age‐related diseases. Extrinsic aging is subdivided into two types: outside effects, like exposure to the sun and UV light, cause extrinsic aging, whereas intrinsic aging involves smooth, dry, and almost wrinkle‐free skin [73].

In humans, these elevated levels of inflammation result in diminished adaptive immunity when subjects advance into old age. Studies on varicella‐zoster virus responses in aged skin identified an increased production of pro‐inflammatory cytokines and innate inflammatory pathways with a diminished infiltration of CD4+ and CD8+ T cells [74]. This accelerating chronic low‐grade inflammatory state during aging accelerates the aging process by amplifying oxidative stress, DNA damage, and stem cell aging. Type 2 inflammation, a type of inflammation that has been associated with aging and the function of the immune system, is very well documented [75]. Reports have suggested that chronically low levels of pro‐inflammatory factors secreted by senescent cells may drive skin immunosenescence and inflammation, possibly linked in a mutual regulatory relationship.

According to scientists, the delay of skin immunosenescence may provide an efficient treatment for inflammatory skin conditions [76]. Aging contains a series of interlinked mechanisms, including proteostatic failure, impairment of autophagy, mitochondrial dysfunction leading to enhanced oxidative stress, stem cell depletion, telomere shortening, cellular senescence with its consequent SASP, epigenetic changes, DNA damage followed by genomic instability, and nutrient signaling incompetence. Most of these processes often lead to increased inflammation, coined “inflamm‐aging.” The mechanisms driving the aging process are multiple, including proteostatic dysfunction, inhibition of autophagy, mitochondrial dysfunction leading to increased oxidative stress, stem cell depletion, telomere shortening, senescence with associated SASP or senescence‐associated secretory program, epigenetic changes, DNA damage, and genomic instability with defective nutrient signaling. This oftentimes results in increased inflammation as part of the so‐called “inflamm‐aging” phenomenon.

**FIGURE 5:**
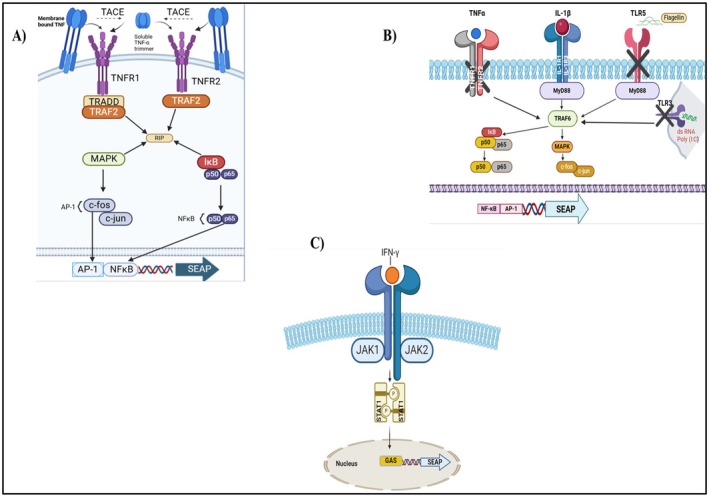
The signaling pathways for various reporter cell lines include: (A) HEK‐Blue TNF‐α signaling pathway, (B) HEK‐Blue IL‐1β signaling pathway, and (C) THP1‐Blue NF‐κB signaling pathway.

Nicotinamide is a member of the vitamin B3 family [77] and has a significant role in anti‐aging using skincare products and cosmetics [78]. Nicotinamide, also part of coenzymes like NAD+, NADH, NADP+, and NADPH, shares the same vitamin properties as nicotinic acid; however, its pharmacological effects and side effects differ. In contrast to nicotinic acid, nicotinamide does not lower cholesterol levels or lead to flushing. Adding nicotinamide as a vital nutrient can have positive effects on overall health and skin. It is unclear whether the efficacy of nicotinamide is its direct effect or its indirect effect acting as a precursor of other active metabolites [78, 79]. It has anti‐inflammatory properties due to the strong downregulation of mRNA for TNF‐α, IL‐1β, and IFN‐γ. The latter, when induced on reporter cell lines, results in the induction of secondary inflammation, which Quanti Blue quantifies (https://www.invivogen.com/hek‐blue‐tnfa) (as illustrated in Figure 5A–C). *Ginkgo biloba* extract functions as an antioxidant and anti‐inflammatory agent by inhibiting the NF‐κB transcription factor and pro‐inflammatory cytokines, such as TNF‐α, IL‐1α, and IL‐6 [80].

Herbal essential oils serve as broad‐spectrum antimicrobial agents widely utilized in cosmetics due to their antibacterial and anti‐inflammatory properties [81]. Proanthocyanidins B1, B2, and C1, found in grape seed oil, are antioxidants that scavenge free radicals. This oil exhibits anti‐carcinogenic, anti‐allergic, anti‐inflammatory, antibacterial, and antiviral properties. Additionally, grape seed oil inhibits NF‐κB activation, and its anti‐inflammatory effects contribute to the suppression of pro‐inflammatory cytokine secretion [82]. *Cocos nucifera* contains lauric acid monoester, an antibacterial compound effective at disrupting microbial cell membranes. Lauric acid, also found in human sebum, is particularly potent as an antibacterial saturated fatty acid. Thyme essential oil contains active components that exhibit anti‐inflammatory, analgesic, and bacteriostatic properties [83]. Sunflower seed oil, which is rich in linoleic acid methyl ester and linolenic acid methyl ester, suppresses the expression of inflammatory mediator genes in cells (Zhao 2012). The methanol extract of tea camellia seed oil, containing polyphenols and squalene, demonstrates significant anti‐inflammatory activity by inhibiting nitric oxide production. Other oils, such as *Baicaojing, Ocimum gratissimum*, cinnamon oil, and star anise oil, are also rich in chemical components that notably inhibit nitric oxide release [84].

Sacha inchi oil, which is rich in tocopherols and sterols, inhibits bacterial growth and exhibits antibacterial and anti‐inflammatory properties. When applied topically, it creates a protective sebum film on the skin, shielding it from bacteria and enhancing its antibacterial and anti‐inflammatory effects [85]. Cinnamon oil, rose oil, and sweet orange oil are recognized for their high antibacterial activity, which may reduce dependency on antibiotics [86]. Various herbal formulations and their applications in the cosmetics industry showcase their multifaceted uses in skincare, haircare, and wound healing (as shown in Table 4). Each formulation leverages the unique properties of natural ingredients to address specific skin concerns, from moisturizing and anti‐aging to anti‐inflammatory and antimicrobial actions.

**TABLE 4:** Herbal formulations and their use in the cosmetics industry.

Sr. No.	Common name of sources	Scientific name	Family	Formulation	Use in the cosmetic industry	References
1	*Aloe Vera*	*Aloe*	Asphodelaceae	Gel and moisturizing cream	Moisturizer, soothing agent, anti‐inflammatory properties	[87]
2	Rosemary	*Rosmarinus*	Lamiaceae	Ointment	Antioxidant, anti‐inflammatory	[88]
3	Lavender	*Lavandula*	Lamiaceae	Antiseptic cream	Wound healing, calming scent, and antiseptic properties, used in skincare and aromatherapy	[89]
4	Tea tree oil	*Melaleuca*	Theaceae	Cream, gel	Treating wounds, burns, and insect bites, antibacterial, antifungal, and effective for acne treatment	[90]
5	Coconut oil	*Cocos nucifera*	Arecaceae	Oil, cream	Moisturizer, hair conditioner, antibacterial properties	[91]
6	Jojoba oil	*Simmondsia*	Simmondsiaceae	Serum and mask	Reduce oxidative stress and inflammation emollient, closely resembles the skin's natural oil and balances moisture	[92]
7	Shea butter	*Butyrospermum*	Sapotaceae	Skin moisturizer creams, emulsions, and hair conditioners	Deep moisturizer, rich in vitamins, helps with skin elasticity	Ayanlowo et al. 2021
8	Witch hazel	*Hamamelis*	Hamamelidaceae	Ointment	Astringent reduces inflammation and is used in toners and cleansers	[93]
9	Green tea	*Camellia*	Theaceae	Emulsions, masks, scrubs, and cleansers	Antioxidant, anti‐aging properties, soothes skin	[94]
10	Hyaluronic acid	*Hyaluronan*	N/A	Creams, gels, mouthwashes	Hydration, plumps skin, reduces wrinkles, reduces bad odor	[95]
11	Ginseng	*Panax*	Araliaceae	Face wash, mask, toner	Energizing promotes circulation, used in anti‐aging products	[96]
12	Argan oil	*Argania*	Sapotaceae	Cream, serum, conditioner	Nourishing hydrates skin and hair, rich in fatty acids	[91]
13	Cucumber	*Cucumis*	Cucurbitaceae	Masks and toners	Hydrating, soothing	[97]
14	Grapeseed	*Vitis*	Vitaceae	Cream, oil	Lightweight moisturizer, rich in antioxidants	[98]
15	Chocolate	*Theobroma*	Malvaceae	Mask, scrub	Hydrating, rich in antioxidants, and used in masks and scrubs	[99]
16	Neem	*Azadirachta*	Meliaceae	Face wash, cream	Antibacterial, antifungal, used in acne treatments	[100]
17	Chamomile	*Matricaria*	Asteraceae	Gel, cream	Calming agent, anti‐inflammatory, used in skin treatments	
18	Papaya	*Carica*	Caricaceae	Facial kit, face wash	Exfoliating contains enzymes that remove dead skin cells	[101]
19	Bamboo extract	*Bambosa vulgaris*	Poaceae	Cream, body lotion	Strengthens hair, provides silica for skin elastic	[102]
20	Pot marigold	*Calendula*	Asteraceae	Ointments, cream	Soothing, anti‐inflammatory and is used in creams and ointments	

## 3 Synergistic Effects of Herbal and Chemical Cosmetics

The term “synergistic effect” refers to an influence that is produced by at least two substances, with the generation of a higher effect than what one of them could have possibly produced alone. This is attributed to the interaction of chemical substances or biological structures in a manner that gives a more dramatic collective effect than the separate contributions. For instance, the effects of tobacco smoke and UV radiation put together are more destructive to skin tissues than if they are used in isolation [103]. Partially, these complex biological processes that come with aging can be alleviated by several bioactive compounds present in different plant species. The newest research shows the synergistic effects of these compounds in enhancing physiological functions and attenuating aging and age‐related diseases [104]. Table 5 summarizes such synergistic effects in skin health applications.

**TABLE 5:** Synergistic effects of herbal and natural compounds in anti‐aging and skin health applications.

Collegial compounds	Activities	Outcomes	References
*Aloe barbadensis miller* extract with Trimethylglycine	Hydrating, anti‐inflammatory, and moisturizing	*Aloe barbadensis* leaf extract, in combination with trimethylglycine, is superior through in silico, in vitro, and clinical studies compared to moisturizing and anti‐inflammatory activity. For instance, it strongly increased the aquaporin 3 (AQP3) levels of the skin, thus enhancing hydration and barrier function	[105]
*Potentilla fruticosa* combined with EGb761	Protecting from oxidative stress	Results of the combined treatment of *Potentilla fruticosa* with EGb761 were synergistic in antioxidant assays, with isorhamnetin being a key mediator. Mechanisms, such as interactions between phytochemicals, enhance their capacities as antioxidants	[106]
*Angelica sinensis* combined with *Astragalus membranaceus*	Protecting skin cells from oxidative stress and inflammation	Improved many outcomes related to the liver and kidneys of aging‐induced rats. Hence, *Angelica sinensis* is proven effective for anti‐aging and can be used for the deterioration of liver and kidney function in aged populations	[107]
Curcumin combined with aged garlic extract	Protecting from oxidative stress	Curcumin and aged garlic extract appear to manifest potential for an impact on cancer and age‐related diseases through their antioxidant activities that might interact subsequently to neutralize some side effects of monotherapy drug treatments	[108]
Red ginseng combined with velvet antler	Antioxidation defense enhancement and inhibition of signaling pathways	The extracts of red ginseng and velvet antler combination will protect the skin by strengthening antioxidants and blocking MAPK/AP‐1/NF‐κB and caspase pathways in UVB‐exposed cells and mice	[109]
The Chinese medicine (TCM) *Panax ginseng* combined with *Polygonatum cyrtonema, Epiphyllum oxypetalum, Nelumbo nucifera* and *Osmanthus fragrans*	Antioxidation, anti‐senescence, DNA protection, collagen production, regulation of the x pathway	The combinations demonstrated high anti‐aging and anti‐senescence potential both in silico predictions and in vitro validations. The 5 TCM combination showed superior effects in reducing senescence markers and DNA damage in the fibroblasts and enhancing collagen production besides supporting dermal regeneration	[110]
Combination of *Linum usitatissimum* , *Silybum marianum* , *Cynara scolymus* and *Pistacia lentiscus* extracts on the multiple aging biomarkers	Anti‐oxidation, anti‐aging	The synergistic combination complex demonstrates the potential for acting as a multi‐aging hallmark simulator: it enhances cellular lifespan in cellulo by reducing oxidative stress and boosting antioxidant defenses	[111]
Combination of Carrot extract and Marigold extract in lipid nanocarriers	Anti‐inflammatory effects	The in vivo studies revealed that the developed formulations possessed higher efficiency than a commercial formulation in anti‐inflammatory activity since they obtained stronger inhibitory effects on pro‐inflammatory cytokines, including IL‐1β and TNF‐α	[112, 113]
*Aloe barbadensis miller* combined with curcumin	Fibrinolytic action, anti‐oxidation	A study shows that Turmeric and *Aloe vera* , the supplement agents, form a good combination that not only reduces the burning sensation of the patient to spicy food but also becomes useful in reducing fibrosis thereby allowing greater mouth opening better than applying injection Triamcinolone and hyalase and Triamcinolone ointment alone in patients of Oral submucosal fibrosis	[114]
Combination of *Centella asiatica* extract transfersomes and rosemary essential oil nanoemulsion against	Collagen preservation, oxidative stress reduction	Nanoemulsion, gel containing *Centella asiatica* transfersomes and rosemary essential oil, enhances topical drug delivery with anti‐aging effects. Specifically, the nanoemulsion exhibited the potential to potentially inhibit UVB‐induced wrinkles significantly through the inhibition of lipid peroxidation, expression of MMP, and the stimulation of the TGF‐β/Smad pathway and type I collagen synthesis, therefore improving skin histology	[115]
*Acori Gramineri Rhizoma* and *Angelicae Tenuissimae* Radix combined with *Magnoliae Cortex*	Anti‐fungal	The study investigated the synergistic effect of *Acori Gramineri Rhizoma* and *Angelicae Tenuissimae* Radix combined with *Magnoliae Cortex*, which inhibits the growth of *C. albicans*	[116]
Synergistic Effect of *Acacia senegalensis* and *Kigelia africana* on Vaginal *Candida albicans*	Anti‐fungal	The result showed that the activities against *Candida albicans* were strong and more potent by *Acacia senegalensis* compared to a combination with *Kigelia africana* that was rather more antagonist than synergistic toward the yeast	[117]
*Fridericia caudigera* combined with *Cuspidaria convoluta*	Antibacterial, anti‐fungal	The combined *F. caudigera* with *C. convoluta* extracts effectively inhibited pathogenic bacteria growth at concentrations lower than required when these extracts are used alone. This activity could be attributed to some of the chemical compounds that have been previously discovered in these extracts, such as flavones. Both the extracts and their combinations They were non‐genotoxic and non‐toxic at their therapeutic concentrations	[118]
Curcumin combined with chlorogenic acid and/or catechin	Anti‐inflammatory	Phytoactives combining plant extracts that provide curcumin, chlorogenic acid, and/or catechin, are used for improved strength and endurance, significantly reduced cortisol, increased hydrolysis of adenosine triphosphate (ATP), enhanced blood flow, fast recovery from sports injury, better weight and fat loss as compared with single actives, increasing and strengthening face muscles.	[119]
Combination of four flavonoid compounds including naringin, hesperedin, hesperitin, and neohesperidin with *Saccharomyces Cerevisiae* BY4742	Anti‐aging, anti‐inflammatory, anti‐oxidation	It was found that when combined with hesperetin, neohesperidin also notably decreased the buildup of ROS in yeast. These findings indicate that neohesperidin, a citrus flavonoid, could be a promising natural candidate for combating aging. Exploring how neohesperidin interacts with other flavonoids to enhance yeast lifespan is an intriguing avenue for future research on the anti‐aging properties of citrus	[120]

These studies demonstrate the potential of plant‐based products, mainly when integrated with other phytocompounds and chemical cosmetics derived from plants, to reduce signs of aging on the skin. Such effects stem from oxidative stress and inflammation, collagen breakdown, DNA damage, and bacterial infections [115]. Synergism makes herbal extracts potent for age‐related and skin‐related conditions. *Centella asiatica* L. Urban (CA), referred to as “Pegagan” in Indonesia, is widely utilized in traditional Indonesian medicine [115]. This study demonstrates that lipid‐based nanocarriers, specifically CA transfersomes and rosemary essential oil (REO) nanoemulsion, work together as effective topical drug delivery systems with anti‐aging benefits. Their combined application can improve UVB‐induced skin damage by reducing wrinkle formation and enhancing skin structure through the modulation of key biological pathways, including downregulating lipid peroxidation and MMPs while promoting the TGF‐β/Smad pathway and type I collagen synthesis [115, 121].

## 4 Nano‐Formulations for Reducing Skin Aging

Over the past decades, the skincare industry has seen a transformation into nanotechnology formulation, especially in the development of advanced anti‐aging nanoformulations. Nano‐formulations offering significant advancement in anti‐aging skincare enhance the delivery, stability, and efficacy of active ingredients. Utilizing different nanocarriers such as liposomes, niosomes, ethosomes, and polymeric nanoparticles, these products can more effectively reduce wrinkles, improve skin elasticity, and promote overall skin rejuvenation over conventional skincare products. Conventional skin cancer treatment includes surgery and chemotherapy, but many of the chemotherapeutic agents used have unfavorable side effects [122, 123]. The use of nanoemulsion in cosmetics (sunscreen products) is very appealing because its droplet size is < 100 nm, making it more stable and capable of preventing creaming, sedimentation, or coalescence [124]. Additionally, it can increase the solubility of an insoluble active ingredient in water. Because of its low viscosity, clear appearance, and large surface area, nanoemulsions effectively transfer active ingredients to the skin, boosting the sunscreen product's performance. Arianto et al. developed a nanoemulsion sunscreen featuring 3% avobenzone, 7.5% octyl methoxycinnamate, and soybean oil, resulting in a sun protection factor (SPF) value of ~21.6. This was significantly higher than the SPF value of around 15.1 of the sunscreen emulsion formulation [125].

Similarly, Afornali et al. used human skin where a triple‐active nanoemulsion (microencapsulated retinol plus additional actives) generated a far stronger regulation of genes linked to the prevention of skin aging, either microencapsulated retinol alone or tretinoin cream [126]. This results in improved permeability into the aged skin and increased bioavailability. Apart from that, they are biodegradable, non‐toxic, and biocompatible, which could result in fewer side effects than most conventional remedies [127]. Nano‐formulations address the fundamental causes of skin aging, which relate to enhanced penetration through the skin and delivery of antioxidants [128]. Furthermore, these advanced formulations not only provide the benefits of enhanced penetration, stability, and controlled release of active ingredients for skin rejuvenation, but also promote moisture retention and UV protection. Additionally, when combined with sunscreens and antioxidants, these treatments enhance penetration, stability, and safety, effectively preventing and treating photoaging from UV exposure. Besides, nano‐formulations enhanced the solubility, bioavailability, and stability of active ingredients, thus controlling treatment release in an increasingly sophisticated manner with minimal harmful side effects [129, 130].

In contrast, a recent review notes that nanoemulsion‐based gels can load more active ingredients, penetrate deeper, and even exhibit a lower risk of skin irritation compared to standard gels [123]. These examples concretely support the benefits of nano‐formulations and also boost anti‐aging effects by targeting cellular pathways and protecting the skin from external stressors, offering significant benefits in combating skin aging [123, 131].

## 5 Clinical Evidence of Herbal Products for Anti‐Aging

Skin aging is characterized by thinning of the dermis, loss of collagen, decreased elasticity, and the formation of wrinkles [132]. Numerous botanical extracts have been investigated, such as *Panax ginseng* , Ashwagandha ( *Withania somnifera* ), turmeric ( *Curcuma longa* ), *Centella asiatica* , green tea, *aloe vera* , etc., for their anti‐aging effects on skin in human studies. *Panax ginseng* has gained attention for its potential benefits in skin health, particularly through an enzyme‐treated red ginseng powder containing concentrated ginsenosides [132]. A single‐centre randomized, double‐blind, placebo‐controlled trial involved 98 healthy women aged 40 to 60 with moderate photodamage, of whom 78 completed the study (39 receiving ginseng and 39 receiving placebo). Participants were administered 750 mg of the ginseng powder or placebo capsules three times daily over a 24‐week period. Results indicated that the ginseng group experienced a significant reduction in mean eye‐wrinkle roughness and overall photodamage score compared to the placebo group. Furthermore, skin elasticity and moisture levels showed improvements, with enhanced elasticity and decreased trans epidermal water loss. While arithmetic roughness (R5) did not manifest significant changes, both average roughness (R3) and investigator assessments demonstrated favorable results. The mechanisms by which ginseng exerts its effects include the action of ginsenosides as known antioxidants, which may contribute to collagen stimulation. Specifically, the study authors suggest that the ginseng powder upregulated dermal type I collagen and skin ceramides, with prior research indicating increases in collagen I expression and inhibition of filaggrin degradation, leading to improved skin hydration through enhanced ceramide synthesis. Additionally, ginseng is recognized for its anti‐inflammatory and antioxidant activities, which can shield against UV‐induced collagen breakdown. Importantly, the enzyme‐treated red ginseng powder was well tolerated throughout the study, with no clinically significant issues [132].

Ashwagandha ( *Withania somnifera* ) randomized, double‐blind, placebo‐controlled trial investigated a topical lotion containing 8% standardized root extract of Ashwagandha (KSM‐66) for improving skin quality in 56 healthy adults with photoaged skin. After 60 days of once‐daily application, results showed that the Ashwagandha treatment significantly outperformed placebo in global physician‐assessment scores for skin quality, with total photoaging scores decreasing by 74.7% in the Ashwagandha group compared to 48.7% in the placebo group (*p* < 0.0001). Improvements in barrier function and elasticity were also notable, with significant reductions in trans epidermal water loss and increases in skin hydration. Although the study did not directly measure the underlying mechanisms, it is suggested that the withanolides and antioxidants in Ashwagandha may play a role in reducing inflammation and oxidative stress, thereby enhancing collagen integrity. No significant adverse effects were reported between groups [133].

Turmeric ( *Curcuma longa* ) and its active compound, curcumin, have shown potential in dermatology for various skin conditions, including photoaging. Despite the lack of dedicated human randomized controlled trials (RCTs) directly targeting skin photoaging, curcumin's antioxidant and anti‐inflammatory properties suggest benefits. A systematic review highlights preliminary evidence indicating improvements in skin clarity and redness when using curcumin‐based formulations [134]. Mechanistically, curcumin scavenges reactive oxygen species and inhibits pro‐inflammatory pathways like NF‐κB, offering protection to collagen and elastin from UV damage, although this is primarily shown in vitro. Overall, while turmeric/curcumin is generally well tolerated, and skin studies have not reported serious adverse effects, the need for large‐scale anti‐aging trials remains evident.

*Centella asiatica* was utilized in the form of its extracts, notably asiaticoside and madecassoside. A 2020 meta‐analysis encompassing five double‐blind randomized controlled trials with approximately 172 Asian women highlighted the efficacy of topical *C. asiatica* compared to placebo and active comparators. The studies showed significant improvements in wrinkle appearance, with treatments such as asiaticoside lipstick and periocular gel leading to noted reductions in wrinkles over 8 to 12 weeks. Additionally, *C. asiatica* treatments markedly enhanced skin hydration, an effect not observed with tretinoin. Safety profiles revealed that topical extracts of *C. asiatica* were generally well‐tolerated, with fewer adverse events compared to retinoids. Mechanistically, asiaticoside and madecassoside promote dermal collagen synthesis through TGF‐β/Smad pathways and possess antioxidant properties, contributing to increased skin firmness and hydration [135].

*Aloe vera* has been investigated for its skin benefits in a randomized, double‐blind, placebo‐controlled trial involving 64 healthy women aged 30 to 59 years over a duration of 12 weeks. Participants received either an *aloe vera* gel sterol‐enriched yoghurt or placebo daily. Results indicated that the aloe group experienced significant improvements in skin moisture and elasticity, as well as decreased trans epidermal water loss, with an increase in dermal collagen content observed via ultrasound imaging. Although the study found that the F3 index, associated with skin “fatigue,” increased, suggesting enhanced skin quality akin to younger skin. The mechanism is linked to aloe sterols, which stimulate collagen and hyaluronic acid production in dermal fibroblasts, thereby supporting skin matrix synthesis and hydration. Importantly, there were no adverse effects related to the aloe sterol supplementation, and all participants tolerated it well [136]. According to Naeimifar et al., a 12‐week open‐label clinical evaluation assessed the effects of a topical cream containing saffron (*Crocus sativus L*.) extract combined with avocado oil in 20 healthy volunteers. Results showed that 45% of participants experienced at least a one‐grade improvement in overall facial wrinkles, as measured by a 5‐point Global Aesthetic Improvement Scale. The active components in saffron, crocins and safranal, are potent antioxidants that may promote collagen production and protect skin from oxidative damage. Importantly, no allergic reactions or irritations were reported, indicating the formulation was well tolerated [137].

Pomegranate ( *Punica granatum* ), a randomized, double‐blind, placebo‐controlled trial, investigated the effects of a proprietary pomegranate and peel extract (ellagic acid, punicalagins, punicic acid) formulation known as Grantria. Sixty healthy adults received either 300 mg/day of Grantria for 60 days. Results showed that the Grantria group experienced significant improvements, including reductions in crow's feet, forehead wrinkles, and UV‐induced pigmentation, along with enhanced skin radiance, moisturization, elasticity, and firmness. Although 97.5% of Grantria users reported brighter, more hydrated skin, safety assessments indicated no adverse events. The pomegranate's polyphenolic antioxidants are believed to play a key role in these benefits by neutralizing oxidative stress and supporting collagen health [138].

Amla ( *Emblica officinalis* ) and lingonberry extract beverage on skin health in 99 healthy women over 12 weeks. Participants were divided into low‐dose and high‐dose groups, receiving 25–30 mg and 50–60 mg of the extracts, respectively. Results showed significant dose‐dependent improvements in skin elasticity (primary endpoint, *p* < 0.01) and secondary outcomes such as dermal thickness, stratum corneum hydration, and wrinkle depth (*p* < 0.001) in the active groups. The beneficial effects are attributed to the high antioxidant content of Amla, particularly vitamin C, polyphenols, and tannins, which protect collagen and elastin, while lingonberry contributes additional antioxidants like anthocyanins. Additionally, the study reported no adverse events, suggesting good tolerability of the formulations [139].

Green tea, A study investigated the effects of green tea polyphenols on moderate photoaging through a combined topical regimen involving 40 women over 8 weeks. Participants received a daily application of 10% green tea cream and 300 mg of oral green tea extract twice daily. While no significant differences in clinical photoaging grades were observed, skin biopsies revealed a statistically significant increase in dermal elastic fiber content (*p* < 0.05) in the green tea group, suggesting a biological anti‐photoaging effect not reflected in surface assessments. Green tea catechins are known for their strong antioxidant and anti‐inflammatory properties, which help to combat UV‐induced damage by inhibiting matrix metalloproteinases (MMPs) and promoting skin repair. The study noted that while mild skin irritation was the primary side effect, no serious adverse events occurred; longer treatment durations may be needed for more visible clinical improvements [140]. Clinical trials have shown that several herbal products can positively affect skin aging in humans. Notable findings include *Panax ginseng* , which significantly reduces wrinkles and improves skin elasticity. Additionally, Withania lotion has been found to enhance scores related to photoaging, while creams containing *Centella asiatica* increase collagen‐associated hydration and reduce periocular wrinkles.

Additionally, green tea polyphenols were found to raise dermal elastin content, and oral aloe sterols improved skin moisture, elasticity, and collagen density. Lesser‐known botanicals like saffron and pomegranate also showed benefits in reducing wrinkles and enhancing skin firmness. The positive effects are largely attributed to the antioxidant flavonoids and triterpenes in these herbs, which protect against UV‐induced free radicals and stimulate collagen and hyaluronic acid synthesis. Overall, these herbal formulations were well tolerated, with minor adverse effects reported infrequently [140]. To illustrate the practical applications of these findings, Table 6 summarizes several marketed anti‐aging products, highlighting their active ingredients, mechanism of action, and benefits.

**TABLE 6:** Marketed anti‐aging products: Ingredients, mechanisms, and benefits.

Product name	Active ingredients	Marketed preparations	Mechanism of action	Benefits	Dosage form	References
Collagen Plus	Hyaluronic acid, vitamin C, collagen peptides	Vital proteins collagen and neocell super collagen	Improves skin suppleness and encourages the generation of collagen	Reduces wrinkles and hydrates skin	Powder	[141], [142]
NeuroBoost	Omega‐3, phosphatidylserine, and *ginkgo biloba*	Nature's abundant superfood for the brain	Increases neuroprotection and blood flow	Enhancement of memory and cognitive support	Pills	[143], [144], [38]
jointEase	Chondroitin, MSM, and glucosamine	Move free, osteo bi‐flex	Promotes cartilage growth and reduces inflammation	Joint mobility and cartilage support	Tablets	[145], [146], [147]
Young Herbalists	Curcumin, ashwagandha, and ginseng	Himalayan ginseng and ashwagandha	Oxidative stress is decreased by the adaptogenic impact	Stress‐relieving and energy‐boosting	Capsules	[113], [148]
AgeDefy Serum	Niacinamide and retinoids	Regular retinol, olay regenerist	Encourages cell turnover and collagen synthesis	Skin rejuvenation and wrinkle reduction	Serum	[149], [150]
Memory Guard	Monnieri bacopa	Alpha‐GPC bacopa plus, alpha brain	Promotes cognitive function and improves neurotransmission	Memory enhancement	Capsules	[151], [152]
Anti‐Aging Blend	L‐carnosine, astragalus, and green tea extract	Herbal code, AMPK activator for life extension	Cell defense and antioxidant activity	Anti‐aging, longevity aid	Capsules	[153], [154]
The Ordinary Buffet	Argireline, hyaluronic acid	The ordinary	Stimulates collagen production and reduces dynamic wrinkles	Skin firming, wrinkle softening, and hydration	Serum	[155], [156]
CoQ10 Vital	Coenzyme Q10 (Ubiquinol)	Qunol ultra CoQ10, doctor's best high absorption CoQ10	Mitochondrial energy production and antioxidant defense	Skin firming, reduced fine lines, and cardiovascular support	Softgels	[157], [158]
Skinceuticals C E Ferulic	Vitamin C (L‐ascorbic acid), vitamin E, ferulic Acid	Skinceuticals	Antioxidant protection against photoaging boosts collagen	Brightening, skin tone improvement, wrinkle reduction	Serum	[159], [160]
Rejuvenol	Vitamin E, alpha‐lipoic acid, and green tea extract	Derma E firming DMAE moisturizer	Antioxidant synergy improves the dermal collagen matrix	Anti‐wrinkle, skin hydration, and skin toning	Cream	[161]

## 6 Safety and Efficacy of Herbal Extracts

Plant materials for safety and quality management are direct concerns that directly link to cosmetic product safety. In India, there are thousands of business enterprises producing plant extracts; however, the lack of national and industrial standards leads to considerable variations in the quality of the products. There are several factors related to this issue. First, differences in growth conditions from one region to another lead to intrinsic differences in plant materials. More importantly, primarily profit‐driven companies may engage in forgery or lower raw materials just to maximize profit. Additionally, the quality of the final product and its cosmetic stability depend significantly on the processing methods employed, which include the extraction, separation, and purification of active ingredients. Ensuring a stable supply of high‐quality plant materials is therefore crucial [162]. Extraction techniques range from traditional soaking, decoction, and steam distillation to modern techniques such as ultrasound‐assisted extraction and supercritical fluid extraction. Liquid extracts are widely utilized in cosmetic formulations because their components are rapidly incorporated, enhancing efficiency while remaining cost‐effective [163]. Solvent choice is determined by active substance polarity; the most polar active substance is extracted with water, and less polar or nonpolar active substances are extracted with ethanol or oils. The re‐dissolvability of the extracts into a more polar solvent might require the inclusion of cosmetic moisturizers, such as propylene glycol or glycerin, for re‐dissolution. There are no standards in the country that would impose the responsibility for quality control. Regulatory bodies need to expedite building great quality management systems, and manufacturing and safety of plant materials have to ensure high standards of raw materials. All requirements of regulations include the risk evaluation of ingredients through full risk assessments, for instance, acute toxicity tests, irritation tests, and sensitization tests, among others, that are experienced across various other consumer demographics results in the provision of safety requirements and formulations, further emphasizing the need for a scientifically sound regulatory framework [164].

However, there are other significant issues with plant extracts, including susceptibility to instability, sensitivity to pH changes, color change, and flocculation. Most of these challenges arose from the intricate composition of the components found in crude extracts. To enhance their stability and, consequently, their overall effectiveness, implementing deep filtration techniques along with the incorporation of stabilizers could be beneficial. In summary, plant extracts in cosmetics must be controlled in quality and subject to full safety evaluation in addition to optimization strategies on stabilization. For the cosmetic industry, such will establish standardized regulatory frameworks to harmonize processes and protect consumers. The emphasis on “Focus on Quality and Safety” naturally aligns with the “Safety and efficacy of herbal extracts.” Both concepts underscore the importance of stringent quality control measures in the cosmetic industry, particularly for plant‐based ingredients. By merging these ideas, we can emphasize that ensuring the safety and efficacy of herbal extracts not only requires tracking and authenticating ingredients, such as through blockchain and Fourier Transform Infrared spectroscopy (FTIR) technology, but also highlights the need for innovative testing methods that replace outdated practices like animal testing [165]. This integration recognizes that the integrity of cosmetic formulations hinges on both their quality and the proven safety of their herbal components, ultimately enhancing consumer trust and regulatory compliance.

## 7 Challenges and Future Directions

Recent advancements of emerging plant‐based actives in modern skincare include ingredients like bakuchiol (Psorelea corylifolia), a clinically validated retinol alternative that stimulates collagen without irritation [166], and buckthorn oil, shown to activate fibroblasts and reduce wrinkles via omega‐7 pathways [167]. While these innovations show promise, challenges such as sustainable sourcing ( *Centella asiatica* ) and allergenicity risks (e.g., *chamomile*) require urgent attention [168]. Therefore, interest in sourcing materials that offer advantages in preservation, such as functional oils (e.g., Tamanu), having a structure that mimics skin lipids and repairing the barrier function, is increasing. These are included in such formulations seeking to provide hydration and antioxidant defense (e.g., polyphenols, tocopherols) and also to reduce inflammation; thus, they can expect to be essential in innovations in skincare products in the future [169]. The critical challenges are the ethical harvesting of rare plants (e.g., overcultivation of *Centella asiatica* ) and allergenicity risks (e.g., *chamomile*) [168].

## 8 Integration of Tradition and Science

New approaches, such as microbial fermentation, involve digesting complex plant extracts (e.g., polyphenols and polysaccharides) by microbes into smaller molecules (e.g., phenolic acids and peptides), which increases the potency and skin absorption. Fermented ginseng enhances ginsenoside bioavailability and boosts skin collagen synthesis. The integration of traditional herbal knowledge with modern science has given rise to natural active cosmeceuticals [170]. Artificial intelligence (AI) is also revolutionizing the plant‐based skincare sector. Machine learning allows skin care researchers to analyze large datasets on plant phytochemistry and skin care biology to predict optimal compound synergies. For example, brands like Herbivore Botanicals and Fenty Skin leverage AI for personalized serums that combine hyaluronic acid with adaptogens such as ashwagandha. This approach evaluates complex interactions between plant‐based compounds. These AI‐based advancements may help us find more accurate and personalized formulations, enabling us to bridge the gap between herbal knowledge and modern scientific advancement. The challenges may include batch variability for standardized fermented extracts and ethical concerns over AI replacing traditional herbal knowledge.

## 9 Nanotechnology for Enhanced Delivery

Nanotechnology has revolutionized the field of effectively delivering bioactive compounds, particularly in skincare formulations. Despite the advancements, several challenges must be addressed to fully realize the potential of nanocarriers such as biodegradable liposomes [171].

## 10 Concerns About Stability

One significant challenge is the inherent instability of nano‐sized particles. They may encounter physical barriers, such as aggregation, which can adversely affect their efficiency. Additionally, many herbal compounds degrade from environmental factors like heat and light, posing a risk to their stability [172].

## 11 Market Cost of the Product and Ability for Large‐Scale Use

Creating these sophisticated nano‐formulations can require advanced technologies, including high‐pressure homogenization. This complexity results in higher production costs, making it difficult to scale while ensuring consistent product quality.

## 12 Standardization Issues

Another challenge lies in the variability of plant materials. The quality and quantity of active compounds fluctuate, necessitating standardized sourcing and testing protocols to guarantee product effectiveness and safety [173]. Despite these challenges, there is optimism regarding the integration of nanotechnology and herbal extracts in anti‐aging applications. Recent advancements have sparked endeavors to hyper‐personalize skincare solutions, such as AI‐powered protective skincare systems. Innovations like pH‐sensitive cellulose nanocrystals aim to target inflamed skin more precisely, while sustainable practices in green chemistry and lab‐grown botanicals are paving the way for environmentally responsible product development. While challenges remain, ongoing research focuses on enhancing stability and affordability in production methods without compromising quality. Future developments could lead to biodegradable delivery systems, AI‐driven formulations, and targeted‐release technologies. As the market for effective, natural anti‐aging solutions grows, addressing these issues will become essential for the successful deployment of nano‐herbal formulations, ensuring scientific validation, customer transparency, and sustainability to achieve the ultimate goal of promoting youthful skin.

## 13 Conclusion

The review emphasizes the multi‐factorial aspect of skin aging and the promise of natural‐origin herbal extracts to modulate it. Skin aging is a multi‐factorial process involving intrinsic and extrinsic aging because of genetic, environmental, and lifestyle factors. Aging is characterized by deep changes like collagen degradation, thinning of the epidermis, and increased oxidative stress, which cumulatively give rise to signs of aging. Plant extracts with high concentrations of natural bioactive molecules, such as polyphenols, flavonoids, and essential oils, are highly effective in skincare. They have moisturizing, antioxidant, and anti‐inflammatory properties and enhance the repair of the skin barrier, making them highly sought‐after ingredients in anti‐aging cosmetics. Their ability to modulate multiple factors of skin aging makes them alternative compounds to the traditional chemical agents. The blending of herbal extracts with nano‐formulations enhances their efficacy, stability, and penetration into the skin. The new method addresses the issue of delivering active ingredients to fight skin aging. By improving the bioavailability of such compounds, nano‐formulations are able to increase the effectiveness of herbal extracts on the skin many times. Recent clinical trials that have been conducted have established the effectiveness of herbal supplements in increasing skin hydration and elasticity and removing visible evidence of aging. These trials constitute concrete evidence for the efficacy and safety of herbal preparations for their application in skin care, to warrant their place in contemporary anti‐aging treatment. There also exists increasing demand from consumers for natural and organic skin care products because of health and environmental consequences arising from artificial product use. This is evident in increasing market demand for herbal cosmetics due to the fact that customers require safer and eco‐friendly products during their skincare process. The review indicates current needs for research in fields like the synergistic effect of chemical and herbal constituents, the development of routine extraction methods of standardization, and the identification of new plant sources. In addition, technological developments, especially in nanotechnology and artificial intelligence, are likely to result in more efficient and specific skincare products.

## Author Contributions

D.S.: wrote four sections of the manuscript; Anand K., Arun K., S.K.: conceptualized the manuscript and wrote two sections; A.J.: wrote one section from the manuscript; S.K. and D.K.: design and validate the manuscript; R.N. and H.G.: design and validate and supervised the whole manuscript.

## Conflicts of Interest

The authors declare no conflicts of interest.

## Data Availability

The data that support the findings of this study are available on request from the corresponding author. The data are not publicly available due to privacy or ethical restrictions.
